# Acute oral toxicity of *Pereskia bleo* and *Pereskia grandifolia* in mice

**DOI:** 10.4103/0973-1296.59969

**Published:** 2010-02-13

**Authors:** K. S. Sim, A. M. Sri Nurestri, S. K. Sinniah, K. H. Kim, A. W. Norhanom

**Affiliations:** *Institute of Biological Sciences, Faculty of Science, University of Malaya, 50603 Kuala Lumpur, Malaysia*; 1 *Department of Physiology, Faculty of Medicine, University of Malaya, 50603 Kuala Lumpur, Malaysia*; 2 *Center for Foundation Studies in Science, University of Malaya, 50603 Kuala Lumpur, Malaysia*

**Keywords:** Acute oral toxicity, Cactaceae, *Pereskia bleo*, *Pereskia grandifolia*

## Abstract

*Pereskia bleo* and *Pereskia grandifolia*, belonging to the botanical family Cactaceae, have been traditionally used by the locals in Malaysia for treatment of various ailments. The current study reports the outcome of acute oral toxicity investigation of *Pereskia bleo* and *Pereskia grandifolia*, on ICR mice. No mortalities or evidence of adverse effects have been observed in ICR mice following acute oral administration at the highest dose of 2500 mg/ kg crude extracts of *Pereskia bleo* and *Pereskia grandifolia*. This is the first report on the acute oral toxicity of *Pereskia bleo* and *Pereskia grandifolia* and the findings of this study are in agreement with those of *in vitro* experiments and thus provide scientific validation on the use of the leaves of *Pereskia bleo* and *Pereskia grandifolia*.

## INTRODUCTION

Medicinal herbs have always been used as traditional primary healthcare agents, especially in Asian countries. Over the last 20 years, rapid changes have been observed in the popular use of natural products from plant sources for maintainence of health and for alternative therapy, in Western countries.[1]

*Pereskia bleo* and *Pereskia grandifolia*, commonly known as ‘Jarum Tujuh Bilah’ in Malaysia belong to the botanical family Cactaceae. *Pereskia bleo* can be easily confused with *Pereskia grandifolia* because they are vegetatively similar. However, they can be easily distinguished by the leaves, flowers, and spines. *Pereskia bleo* has thinner, corrugated leaves, and orangish-red flowers, with shorter spines compared to *Pereskia grandifolia*. In contrast, *Pereskia grandifolia* has thicker, uncorrugated leaves, and pink to purplish-pink flowers, with longer and lesser spines.[2]

Both *Pereskia bleo* and *Pereskia grandifolia* have been used as natural remedies in cancer-related diseases, either eaten raw or taken as a concoction brewed from fresh plants. Both are believed to have anti-cancer, anti-tumor, anti-rheumatic, anti-ulcer, and anti-inflammatory properties. They are also used as remedies for the relief of headache, gastric pain, ulcers, hemorrhoids, atopic dermatitis, and for revitalizing the body.[3–5] In Panama, the locals use the whole plant of *Pereskia bleo* to treat gastrointestinal problems,[6] while in India, *Pereskia grandifolia* is used to reduce swelling.[7–8]

Although *Pereskia bleo* and *Pereskia grandifolia* are reported to be used in a large number of Malaysian traditional medicine preparations, there is no published report on the study of acute oral toxicity of both *Pereskia bleo* and *Pereskia grandifolia*. The acute oral toxicity test is the simplest, and often the first toxicity test to be conducted on a sample. A single, high dose of the test sample is given to each experimental animal and the mortality is observed; death within the observation period (usually of 14 days duration), whether caused by natural death or humane killing, is studied.[9] The findings of this study corroborated the need for a safety study on both the *Pereskia* species used for primary health care in Malaysia. Such studies need to be carried out before the continued widespread use of some species provokes long-term and irreversible damage.

Previous cytotoxic investigations on *Pereskia bleo*[5,10–11] and *Pereskia grandifolia*[2] reported that both *Pereskia bleo* and *Pereskia grandifolia* had cytotoxic effects on various cancer cell lines, using *in vitro* cytotoxic activity assays, but did not show cytotoxicity against the normal cell line.[2,11] If this also occurs *in vivo,* the use of this plant, by locals, for cancer treatment, would have scientific support.

## MATERIALS AND METHODS

### Plant sample collection and identification

The fresh leaves of *Pereskia bleo* and *Pereskia grandifolia* were collected from Petaling Jaya, Selangor, Malaysia in September 2006 and February 2007, respectively. They were identified by Professor Dr. Halijah Ibrahim of the Institute of Biological Sciences, Faculty of Science, University of Malaya, Malaysia, and voucher specimens were deposited in the herbarium of the Institute of Biological Sciences, Faculty of Science, University of Malaya, Kuala Lumpur, Malaysia, with voucher numbers of SN01-06 (*Pereskia bleo*) and SN01-07 (*Pereskia grandifolia*).

### Chemicals

Anhydrous sodium sulfate was purchased from the Sigma-Aldrich Company, while Tween 80 and methanol were obtained from the Merck Company.

### Preparation of extracts

The crude extracts were prepared as previously described.[11] Briefly, the fresh leaves of *Pereskia bleo* and *Pereskia grandifolia* were washed, dried, and ground to a fine powder, using a blender. The dried, ground leaves were then soaked in methanol (1.5 L) for three days, at room temperature. The solvent-containing extract was then decantered, dried with anhydrous sodium sulfate, and filtered. The extraction of the ground leaves was further repeated (2x) with methanol (1.5 L each time). The filtrates from each extraction were combined and the excess solvent was evaporated (Buchi, Rotavapor, Switzerland) under reduced pressure, using a rotary evaporator, to give a dark green crude methanol extract.

### Test species

The experiment was performed on healthy ICR mice (five weeks of age, body weight 23-28 g), obtained from the Laboratory Animal Center, Faculty of Medicine, University of Malaya. The female mice were confirmed nulliparous and non-pregnant. The mice were assigned to five dosage groups and one control group with 10 mice (five male and five female) for each test group. The weight variation in the mice used did not exceed ± 20% SD of the mean body weight of each sex. The experimental procedures involving the animals were approved by the University of Malaya Animal Experimental Ethnics Committee [Ethical number: ISB/05/08/2009/SKS (R)] before commencing the study.

### Procedure of acute oral toxicity

The acute oral toxicity of the crude methanol extracts of both the *Pereskia* species were evaluated in mice using the procedure described by the OECD (Organization for Economic Co-operation and Development), with some modifications. The mice were housed in suspended, stainless steel, wire-mesh cages in an experimental animal room. The temperature was maintained at 23 ± 3°C and the relative humidity was 50-60% before and after treatment with the extract. The animal room was artificially illuminated (fluorescent light) with an approximate 12-hour light/ dark cycle. The mice were acclimatized to the laboratory conditions for at least five days prior to commencement of the experiments. The mice were randomly selected for use in the study and marked to provide individual identification. Conventional mouse diets, with unlimited supply of drinking water, were available *ad libitum*, except during the fasting period. The mice were fasted approximately 12 hours prior to dosing, but they had free access to drinking water. Before and after treatment with the extract, the mice were caged in groups by sex and dose levels. The extracts were suspended in a vehicle (10% Tween-80 in distilled water). A stock concentration of 200 mg/ml was prepared and the mice were administrated with 0.2 ml of the extract for every 10 g of mice body weight. The mice were administered with doses of 500, 1000, 1500, 2000, and 2500 mg/kg of extracts. Food was started for the animals approximately three to four hours after dosing. The mice were observed carefully for any signs of toxicity in the first four hours after the treatment period, and daily thereafter for a period of 14 days. Observations for mortality, signs of illness, injury, pain, distress, allergic reactions, changes of outer appearance, behavioral alterations (i.e., ataxia, hyperactivity, hypoactivity), and general stimulation or sedation were conducted twice daily. The observations were recorded systematically; individual records were maintained for each mouse.

## RESULTS AND DISCUSSION

### Extraction yield of *Pereskia bleo* and *Pereskia grandifolia*

Solvent extraction is the most popular method used in sample preparation. The yields from methanol extracts of *Pereskia bleo* and *Pereskia grandifolia* are shown in Table 1. Before extraction, the plant material needs to be dried to avoid the presence of water in the extracts. The percentage of crude methanol extract yield is based on the weight of dried and ground plant materials. Methanol is used as the extraction solvent due to its polarity and its known ability to extract compounds such as, phenolics, flavonoids, and other polar materials.[12]

**Table 1:** Yield of methanol extracts of Pereskia bleo and *Pereskia grandifolia*

Plants	Samples/extracts	Weight (g) (%)
*Pereskia bleo*	Fresh samples	4526.79
	Dried and ground plant material	752.92 (16.63)
	Methanol extract	79.81 g (10.60)
*Pereskia grandifolia*	Fresh samples	410.18
	Dried and ground plant material	37.05 (9.03)
	Methanol extract	9.91 (26.75)

### Acute oral toxicity assessment of *Pereskia bleo* and *Pereskia grandifolia* crude extracts

Investigation of acute toxicity is the first step in the toxicological analysis of herbal drugs.[13] Overall, animal models have a good predictability for human toxicities of around 70-80%.[14,15] Generally, it is possible to get the first hints on complex toxicities by applying *in vivo* methods, as information on some toxic manifestations cannot be assessed by *in vitro* cytotoxicity methods.[16] Toxic manifestations that affect the entire organism such as pain, distress, allergic reactions, changes in outer appearance, behavioral alterations, and general stimulation or sedation can be detected by *in vivo* assays. However, the detection of effects on vital functions (cardiovascular, central nervous, and respiratory systems) is usually not assessed in acute toxicity studies.

Acute oral toxicity was undertaken in the present study to determine the safety parameters of the leaves of *Pereskia bleo* and *Pereskia grandifolia*. Mortality, clinical signs, gross findings, and body weights of mice were observed and measured for 14 days after the oral administration of crude methanol extracts to both *Pereskia sp*. The crude methanol extracts were used in this acute oral toxicity study to ensure that all components in the extract were included.

Table 2 shows the results of the acute toxicity of the crude extracts of *Pereskia bleo* and *Pereskia grandifolia*. For all doses tested for crude methanol extracts of *Pereskia bleo* and *Pereskia grandifolia*, there were no deaths reported. Throughout the 14-day observation period, there were no significant changes in behavior (i.e., ataxia, hyperactivity, hypoactivity) in any of the mice, nor did they produce any variations in the general appearance. They gained weight with no adverse clinical signs of toxicity at any dose.

**Table 2:** Results of the potential toxic effect of the crude extracts of *Pereskia bleo* and *Pereskia grandifolia* in mice

Plants	Dose (mg/kg)											

	0^a^		500		1000		1500		2000		2500	
						
	M	F	M	F	M	F	M	F	M	F	M	F
*Pereskia bleo*	0/5^b^	0/5	0/5	0/5	0/5	0/5	0/5	0/5	0/5	0/5	0/5	0/5
*Pereskia grandifolia*	0/5	0/5	0/5	0/5	0/5	0/5	0/5	0/5	0/5	0/5	0/5	0/5

M = Male ICR mice; F = Female ICR mice;

a control group (treatment without extract);

b Number of animals dead/number of mice used

Traditionally, the aim of the acute oral toxicity study was the estimation of LD_50_. The LD_50_ value — defined as the statistically derived dose, which when administered in an acute toxicity test, is expected to cause death in 50% of the treated animals in a given period — is currently the basis for toxicological classification of chemicals. For a classical LD_50_ study, laboratory mice and rats are the species typically selected. Often both sexes must be used for regulatory purposes.[14]

As no deaths were found for all doses tested for crude methanol extracts of *Pereskia bleo* and *Pereskia grandifolia*, the LD_50_ values of crude *P*. *bleo* and *P*. *grandifolia* extracts were >2500 mg/kg. This indicated that both *Pereskia sp*. did not cause any acute toxicity. According to the chemical labeling and classification of acute systemic toxicity, based on oral LD_50_ values, which were recommended by OECD,[17,18] the crude extracts of both *Pereskia sp*. were assigned to class 5 (LD_50_ > 2000 mg/kg), which was termed as the lowest toxicity class (no label; *unclassified*). Oliver[19] pointed out that (i) the LD_50_ value was not an absolute value, but was an inherently variable biological parameter that could not be described in terms of accuracy, but only of precision, (ii) the LD_50_ value referred only to mortality and was illustrative of no other clinical expression of toxicity.

## CONCLUSION

In view of the increasing popular consumption of medicinal plants as alternative therapy, it is necessary to conduct research to support the therapeutic claims and also to ensure that the plants are indeed safe for human consumption. The present research findings have clearly met the objectives of the study. The result was in agreement with that of *in vitro* experiments, whereby, the crude extracts of *Pereskia bleo* and *Pereskia grandifolia* did not show cytotoxicity against normal MRC-5 cells.[2,11] Based on the outcome of acute toxicity in experimental mice, the crude extracts of both *Pereskia sp*. could be regarded as safe in experimental mice. Further toxicity study over a longer period of time involving detection of effects on vital organ functions would ensure that the plants are safe for human consumption.
